# Facile Solution Synthesis of Red Phosphorus Nanoparticles for Lithium Ion Battery Anodes

**DOI:** 10.1186/s11671-018-2770-4

**Published:** 2018-11-08

**Authors:** Fei Wang, Wenwen Zi, Bao Xun Zhao, Hong Bin Du

**Affiliations:** State Key Laboratory of Coordination Chemistry, School of Chemistry and Chemical Engineering, Nanjing University, Nanjing, 210023 China

**Keywords:** Red phosphorous, Solution method, Lithium ion battery, Anode material

## Abstract

Red phosphorus (RP) has attracted extensive attention as an anodic material for lithium-ion batteries (LIBs) due to its high theoretical specific capacity of 2596 mA h g^− 1^ and earth abundance. However, the facile and large-scale preparation of the red phosphorus nanomaterials via a solution synthesis remains a challenge. Herein, we develop a simple and facile solution method to prepare red phosphorus nanoparticles (RP NPs). PCl_3_ readily reacts with HSiCl_3_ in the presence of amines at room temperature to produce amorphous RP NPs with sizes about 100–200 nm in high yields. When used as an anode for rechargeable lithium ion battery, the RP NP electrode exhibits good electrochemical performance with a reversible capacity of 1380 mA h g^− 1^ after 100 cycles at a current density of 100 mA g^− 1^, and Coulombic efficiencies reaching almost 100% for each cycle. The study shows that this solution synthesis is a facile and convenient approach for large-scale production of RP NP materials for use in high-performance Li-ion batteries.

**Electronic supplementary material:**

The online version of this article (10.1186/s11671-018-2770-4) contains supplementary material, which is available to authorized users.

## Introduction

It has been realized for a long time that fossil fuels are non-renewable, finite, and environmentally harmful. Rechargeable lithium ion batteries (LIBs) with high energy density and long cycle life have stimulated extensive research interest because of their potentials as efficient and cheap energy storage systems [1–3]. The increasing demands for low-cost lithium ion batteries (LIBs) with high energy density and long cycle life call for the development of novel electrode materials [4–7]. The traditional graphite anode, commonly used in lithium ion batteries, is limited with respect to its low capacities (372 mA h g^− 1^) [8, 9]. To address this problem or issue, a great deal of efforts have been devoted to explore and develop alternative anode materials with substantially improved capacity and Coulombic efficiencies [10–17]. Among a wide range of high capacity anode materials, phosphorus and its composites show potential applications due to its low cost, abundance and high theoretical specific capacity (≈ 2600 mA h g^− 1^) [18–22].

Phosphorus has three allotropes, white P, black P, and red P [23]. White P is toxic and chemically unstable, and is not suitable for the application in LIBs. Black P has good thermodynamic stability and conductivity, but the complex preparation process limits its large-scale applications [24–26]. Among these three different allotropes, red P is the most promising candidate [27] for the next generation high-energy anodic materials because of its stability and abundance. However, red P is plagued by its poor electronic conductivity (10^− 12^ S m^− 1^) and drastic volume change (300%) during the lithiation-delithiation process when served as anodes for rechargeable LIBs [28, 29].

To circumvent these impediments, red P has been encapsulated in different types of carbon host materials [30–36] to substantially improve the electrochemical performance of red P anodes for LIBs. For instance, Li et al. significantly improved both lithium storage and sodium storage performance of red P by confining nanosized amorphous red P into a mesoporous carbon matrix (P@CMK-3) via vaporization-condensation-conversion process [37]. Ruan et al. designed a new strategy to embed red P particles into a cross-link-structural carbon film (P-C film) for use as a flexible binder-free anode in LIBs, in order to improve the electronic conductivity and accommodate the volume expansion [38]. Nevertheless, the loading ratio of red P in the composite materials prepared by the vaporization–condensation method is typically low, which is unfavorable for the practical application [39, 40]. To this end, the use of nanoparticles or hollow nanostructures of red P prepared through size control and morphology engineering [41, 42] have been regarded as effective strategies to accommodate the large strain induced by the volume expansion and avoid material pulverization. For example, Chang et al. developed a large-scale synthesis of red phosphorus nanoparticles (RPNPs) through reduction of PI_3_ in iodobenzene by ethylene glycol in the presence of CTAB. The obtained RPNP electrodes exhibited a high-specific capacity, long cycling life, and excellent rate capability as anodes for LIBs [43]. Moreover, Zhou et al. reported a wet solvothermal method to synthesize hollow red-phosphorous nanospheres with porous shells. The obtained hollow P nanosphere electrodes demonstrated high capacities and excellent long cycling performance due to the merits of the porous and hollow structures [44]. Even though several literatures have reported the methods to the large-scale synthesis of red phosphorus, developing a high-yield and low-cost facile method to prepare the red phosphorus is still highly desirable. Particularly, the preparation of the red phosphorus nanomaterial via a solution synthesis remains a challenge.

Herein, we report a facile, rapid, and novel solution-based approach to synthesize RP NPs, employing room-temperature reaction of PCl_3_ with HSiCl_3_ in CH_2_Cl_2_ in the presence of amines. This new solution provides a cost-effective approach for massive production of red phosphorus nanoparticles for use in lithium ion batteries.

## Methods

### Materials

Trichlorosilane (HSiCl_3_) was purchased from TCI. n-Tripropylamine (Pr_3_N) was obtained from Aladdin. Phosphorus trichloride (PCl_3_) was purchased from Sinopharm Chemical Reagent Co. Ltd. Dichloromethane (CH_2_Cl_2_) was dried over CaH_2_ before use. All other chemical reagents were used as received without further purification.

### Synthesis of Red Phosphorus Nanoparticles

In a typical preparation, 0.55 mL of Pr_3_N and 0.5 mL of HSiCl_3_ were added into 20 mL of anhydrous CH_2_Cl_2_. The formed colorless solution was magnetically stirred overnight at room temperature, during which the color turned into light yellow. And then 0.5 mL of PCl_3_ were added to the solution. Red phosphorus nanoparticles (RP NPs) were obtained in several seconds. The products were centrifuged, separated, and washed with anhydrous CH_2_Cl_2_, 1 M HF and distilled water to remove unreacted PCl_3_ and silica.

### Electrochemical Measurements

The electrochemical properties of red phosphorus nanoparticles as anode materials in LIBs were studied by using a 2032 coin cell assembly with lithium metal foils served as counter electrodes. The CR2032 cells were assembled in an argon-filled glovebox (both H_2_O and O_2_ < 0.1 ppm). The working electrode was prepared by mixing active material (RP NPs), conductive graphite and sodium carboxymethyl cellulose (CMC) in a weight ratio of 5:3:2 in deionized water to form a homogeneous slurry, which was then blade-deposited on a Cu foil. After drying at 80 °C for 12 h in vacuum, the foil was cut into disks of 14 mm in diameter. The total mass loading of active materials on the electrode was ~ 0.5 mg cm^− 2^. The electrolyte was 1.0 M LiPF_6_ in a mixture of 1:1 (*v*/*v*) ethylene carbonate/diethyl carbonate (Shenzhen Kejingstar Technology Ltd., China). The charge-discharge profiles of the half-cells were recorded using a Neware battery testing device (Shenzhen, China) at a constant current mode.

### Characterization

Powder X-ray diffraction (PXRD) was carried on a Bruker D8 X-ray diffractometer with a Cu Kα radiation (λ = 1.5418 Å). Scanning electron microscopy (SEM) images and energy dispersive spectroscopy (EDS) spectra (silicon wafers as the substrate) were obtained on a Hitachi field-emission scanning electron microscope (S-4800). Transmission electron microscopy (TEM) and high-resolution (HR) TEM were carried out with a JEM-2100 equipment (Japan). N_2_ adsorption isotherms were collected at 77 K (Micromeritics ASAP 2020 analyzer) after vacuum degassing of the sample at 100 °C for 10 h. Raman spectroscopy (LabRAM Aramis, Horiba, equipped with 633 nm laser) was used to investigate the structure of RP NPs. X ray photoelectron spectroscopy (XPS) measurements were recorded with a PHI 5000 VersaProbe. Thermo gravimetric (TG) analyses were conducted on a simultaneous STA449F3 (Netzche) thermal analyzer under flowing N_2_. The I-V curves of RP NPs were measured using cryogenic probe station Biologic VMP3 instrument (CRX-4K, Lake Shore, USA). Cyclic voltammetry (CV) tests were performed on a CHI650d electrochemical station (Shanghai Chenhua Instruments Inc., China).

## Results and Discussion

### Synthesis and Characterization of RPNPs

**Scheme 1:**
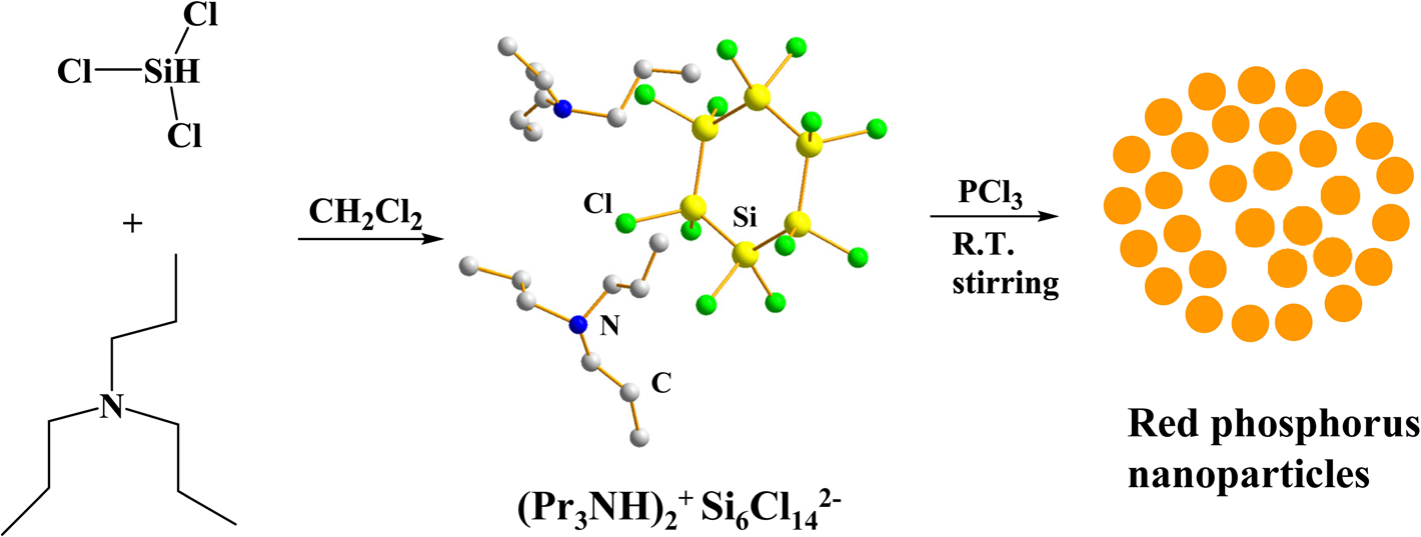
Schematic illustration of the synthesis process of RP NPs

The red phosphorus nanoparticles (denoted as RP NPs) were synthesized via a facile solution method, which is depicted in Scheme 1. We found that phosphorus trichloride (PCl_3_) readily reacted with premixed HSiCl_3_ and tripropylamine (Pr_3_N) in CH_2_Cl_2_ at room temperature to produce orange powders in several seconds. The solution color changed to orange very rapidly when mixing a solution of HSiCl_3_–Pr_3_N–CH_2_Cl_2_ with PCl_3_, indicating the formation of RP NPs (Additional file 1: Figure S1). We postulated that PCl_3_ were reduced by subvalent oligosilane chlorides to form the phosphorus nanoparticles. The oligosilane chlorides were formed by the reaction of HSiCl_3_ with tripropylamine (Pr_3_N) in CH_2_Cl_2_ as results of disproportionation reaction of HSiCl_3_ in the presence of amine catalysts [45–47]. It is noted that the preformed oligosilane intermediates were essential for the occurrence of the reaction. Without amines (Pr_3_N), the reaction of HSiCl_3_ with PCl_3_ could not take place at room temperature. Similarly, the Pr_3_N could not react with PCl_3_ to produce RP NPs at room temperature. The yield of RP NPs, based on the amount of P atoms in PCl_3_, was approximately 38%, which is much higher than the reported literature [43]. Furthermore, this solution-phase approach utilizes relatively low cost PCl_3_ instead of PI_3_ in iodobenzene, which could be more economically and easily scaled up to obtain large amounts of RP NPs. The color of the RPNPs was light orange, differing from the deep red color of commercial RP (Additional file 1: Figure S2).

**Fig. 1:**
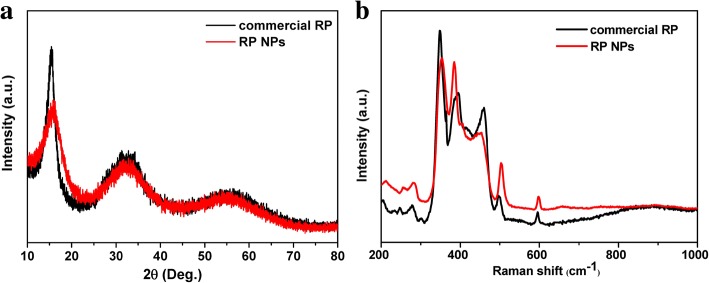
Characterization of RPNPs. **a** XRD patterns of RPNPs and commercial RP. **b** Raman spectra of RPNPs and commercial RP

**Fig. 2:**
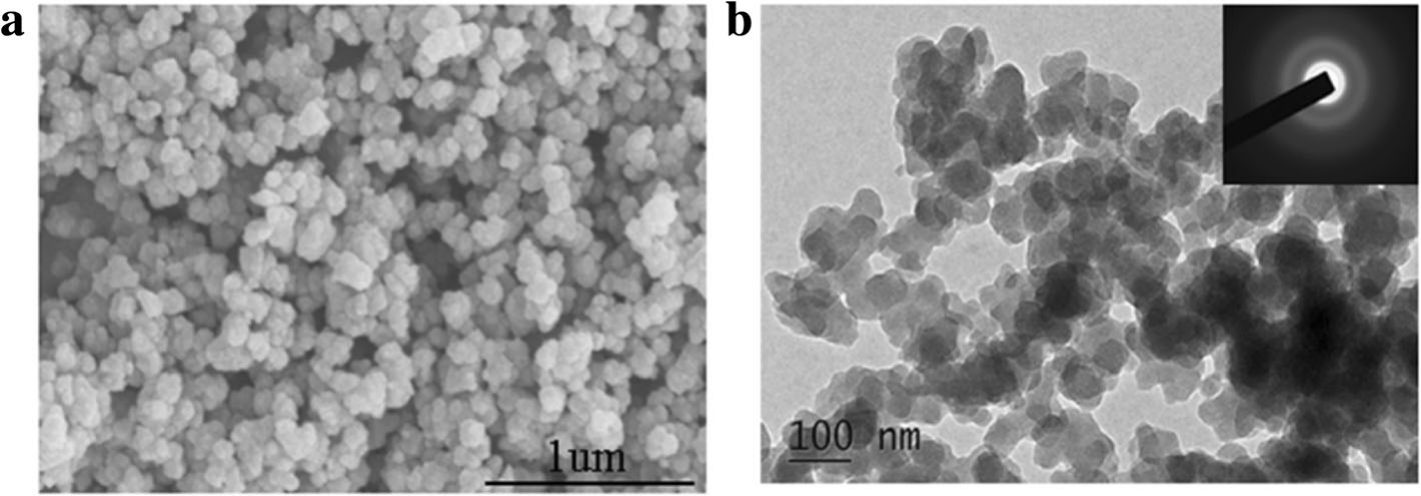
Morphology of RP NPs. **a** SEM images of RP NPs. **b** TEM images of RP NPs. The inset image is the SAED pattern

**Fig. 3:**
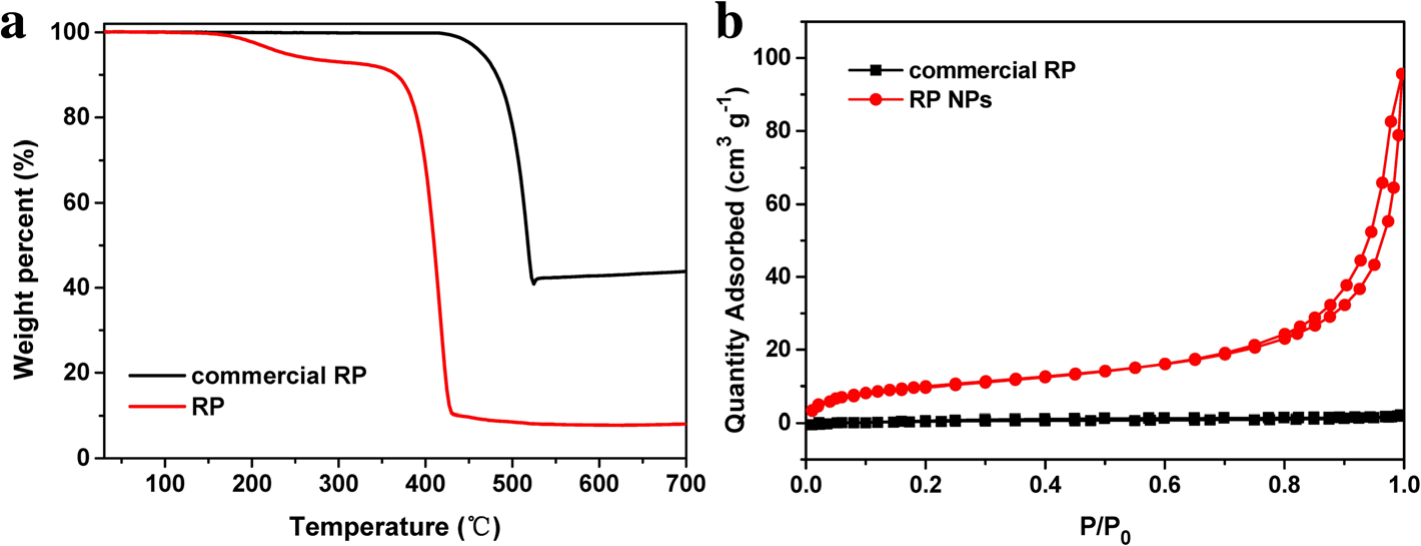
**a** TGA of RP NPs and commercial RP. **b** N_2_ adsorption isotherms of RP NPs and commercial RP

PXRD analysis showed that the product was red phosphorus. As shown in Fig. 1a, the three broadened diffraction peaks at 13–16°, 25–38°, and 47–65°, consistent with the XRD pattern of commercial RP reported in the literature [21, 36]. SEM images show that the majority of RP NPs synthesized exhibited irregular-spherical shape with sizes about 100–200 nm in diameter. The corresponding SAED pattern of RP NPs (inset image of Fig. 2b) revealed that RP NPs were amorphous structure. Raman spectra of RP NPs presented three puckered peaks between 300 and 500 cm^− 1^, which is consistent with the Raman spectrum of commercial RP reported in the literature [36]. The three peaks can be well assigned to the bond bending modes (B1 fundamental mode), bond bending vibrations (A1 symmetric stretch motion), and stretching vibrations (E1 degenerate mode) of amorphous red P (Fig. 1b). The thermal gravimetric analysis (TGA) of RPNPs in Fig. 3a shows a sharp weight loss between 380 and 430 °C under a nitrogen atmosphere owing to the sublimation, whereas commercial RP shows a sharp weight loss between 450 and 500 °C. The observed depression of sublimation temperature of RP NPs may result from high surface-to-volume ratios of nanoparticles [43, 48]. To quantitatively obtain the information of surface area, N_2_ sorption measurements (Fig. 3b) were conducted. The results revealed the Brunauer–Emmett–Teller (BET) surface area of RP NPs was about 37 m^2^ g^− 1^, which is much larger than that of the commercial RP.

**Fig. 4:**
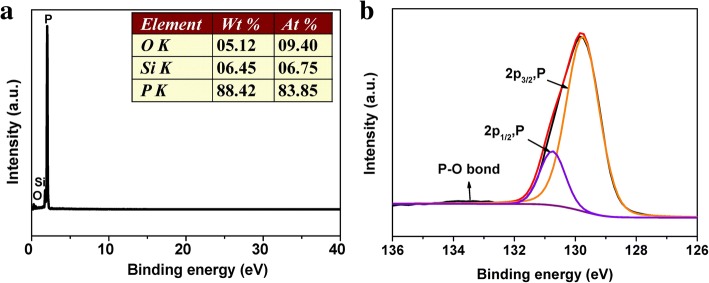
**a** EDS spectra of RP NPs. **b** P 2p XPS spectrum of RP NPs

In order to further characterize the structures, compositions, and chemical states of the prepared RP NPs, energy-dispersive X-ray spectroscopy (EDS) and X-ray photoelectron spectroscopy (XPS) measurements were carried out (Fig. 4). The EDS spectra shows that RP NPs are almost completely composed of elemental phosphorus. XPS survey spectrum (Fig. 4b) further confirms that P is the dominant element. The main peak in P 2p spectrum of XPS could be deconvoluted into two peaks at 129.74 and 130.74 eV, which correspond to the 2p_3/2_ and 2p_1/2_ of P in P–P bond, respectively, according to the previous literature [49, 50]. Moreover, a weak peak at 133.50 eV could be assigned to the P–O bond which was possibly formed through surface oxidation during air exposure. Therefore, the above results indicate the prepared nanoparticles are amorphous red P. In addition, the current-voltage (I-V) curves of RP NPs have been measured, as shown in Additional file 1: Figure S3. The conductivity of the RPNPs is about 1.7 × 10^− 7^ S m^− 1^ (0–2 V), which is 10^5^ times higher than commercial RP (10^− 12^ S m^− 1^).

**Fig. 5:**
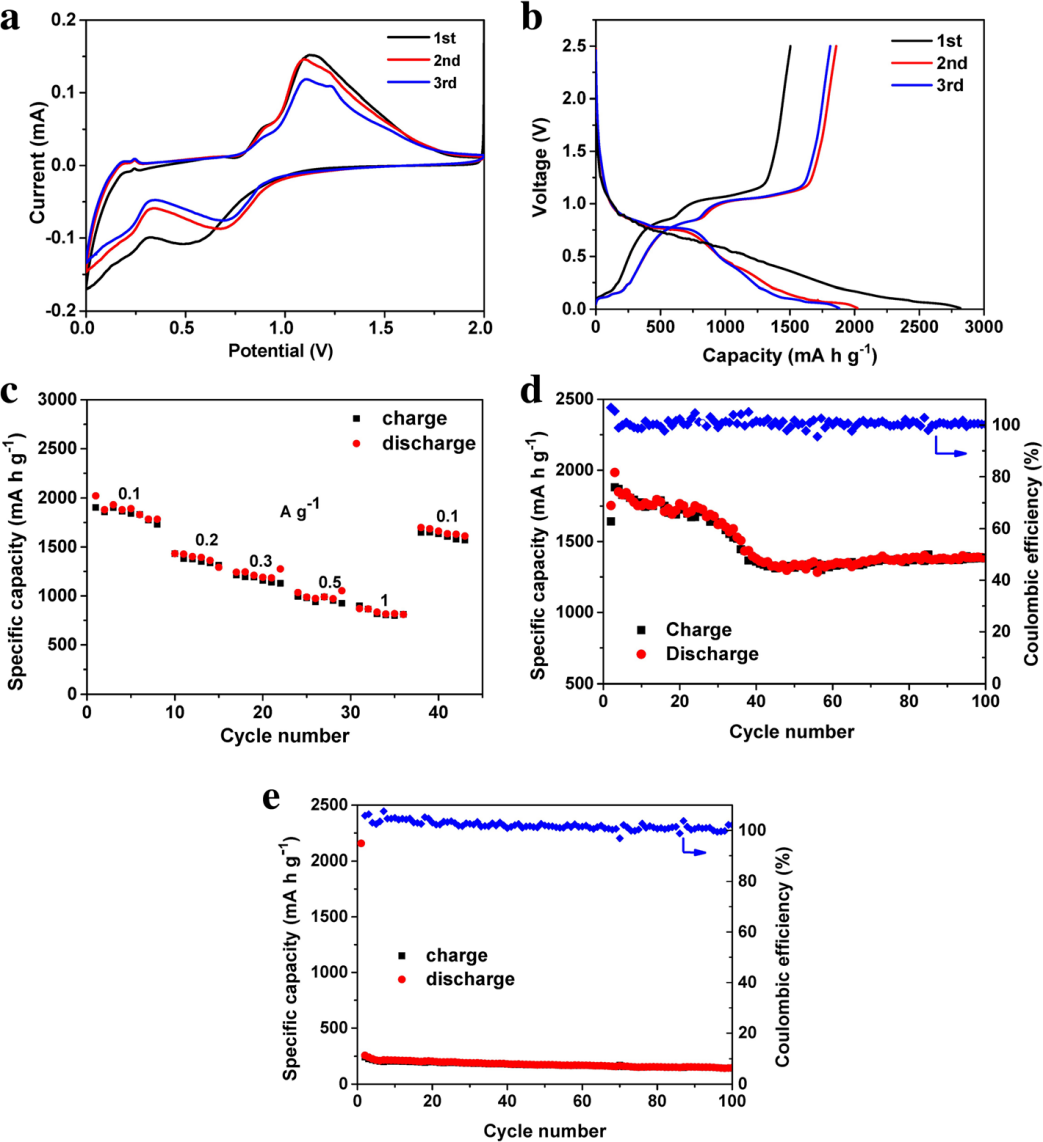
Electrochemical performance of RPNPs. **a** CV curves of the RPNPs. **b** Voltage profiles of the RPNPs. **c** Rate performance of the RPNPs cycled at different current densities. **d** Cycling performance of RPNPs at a rate of 0.1 A g^− 1^. **e** Cycling performance of commercial RP at a rate of 0.1 A g^− 1^

The electrochemical performance of the RP NPs as anode materials in LIBs was tested in CR2032 coin cells using a lithium metal foil as the counter electrode within the operating voltages of 0.01 to 2.5 V. Figure 5a depicts typical CV curves of the RP NPs at a scanning rate of 0.1 mV s^− 1^. There is a broad peak in the first lithiation cycle, which is ascribed to the activation process of inserting Li ions into phosphorus. A couple of redox peaks located at 0.5–0.75 V and 1.0–1.25 V are attributed to the lithiation of P and the delithiation of P–Li alloys [32, 51, 52] respectively. The deviation between the first and the subsequent cathodic curves implies irreversible capacity loss, which could be ascribed to the formation of the solid electrolyte interface (SEI) as well as to the occurrence of side reactions on the electrode surfaces, such as side reactions of defect sites, surface oxygen, and water impurities [36, 37, 53], a commonly observed behavior for LIB anodes. Figure 5b shows the typical discharge-charge voltage profiles of the RP NPs electrode for the first 3 cycles at a current density of 0.1 A g^− 1^. The apparent short discharge and charge voltage plateaus at around 0.7 V and 1.1 V are due to the lithiation and de-lithiation of RP NPs components, respectively, which agree well with the CV results. The electrode delivered a specific discharge and charge capacities of 2818 and 1641 mA h g^− 1^, respectively, for the first cycle, giving a first Coulombic efficiency of 58.2%. The decreased charge capacity could be attributed to the irreversible formation of the SEI membrane. It is noted that the Coulombic efficiency of RP NPs then quickly increased to 100% after the second cycle. The RP NPs exhibited an obvious capacity decay in the first 3 cycles. The irreversible capacity in the first few discharge-charge steps resulted from the decomposition of the electrolyte, which caused the formation of SEI on the electrode surface and the consumption of Li-ion. Moreover, the nanoparticles possess a high surface area in contact with the electrolyte solution, which would result in more side reactions, lowering the initial Coulombic efficiency in the first cycle [54].

The typical rate and long-term cycling stability performance of the RP NPs electrode are shown in Fig. 5c, d, respectively. RPNPs delivered the specific charge capacities of 1801, 1430, 1245, 1227, 1184, and 871 mA h g^− 1^ at the rates of 0.1, 0.2, 0.3, 0.5, and 1 A g^− 1^, respectively. The electrode showed good rate reversibility, with the specific discharge capacity recovered to the initial value when the current density returned to 0.1 A g^− 1^ after cycling at high current densities. The RP NPs finally maintained a high reversible discharge capacity of 1380 mA h g^− 1^, i.e., the retention of 89.1%, after 100 cycles with Coulombic efficiencies close to 100% throughout the measurements. Compared to commercial RP (Fig. 5e), RP NPs showed much improved long-term cycling stability.

## Conclusions

In summary, we developed a new facile solution-phase approach to synthesis red phosphorus nanoparticles through the reaction of PCl_3_ and HSiCl_3_ in the presence of amines under the ambient environment. The RP NPs exhibited much better electrochemical performance with high reversible capacity and long-term cycling stability than commercial RP when served as anodes for rechargeable lithium ion battery. The RP NPs electrodes maintained a high reversible discharge capacity of 1380 mA h g^− 1^ (retention of 89.1%) after 100 cycles, with a Coulombic efficiency close to 100% for each cycle. This simple preparation method paves the way for cost-effective production of RP NPs as high-performance anodes for lithium-ion battery industry.

## Additional file

Additional file 1: **Figure S1.** The reaction process of RPNPs via the solution synthesis. **Figure S2.** Optical images of RPNPs and commercial RP powders. **Figure S3.** I-V curves of RPNPs. Figure S4. SEM images of commercial RP. (DOCX 631 kb)

## Abbreviations

CH_2_Cl_2_: Dichloromethane
CV: Cyclic voltammetry
EDS: Energy dispersive spectroscopy
HSiCl_3_: Trichlorosilane
PCl_3_: Phosphorus trichloride
Pr_3_N: n-Tripropylamine
PXRD: Powder X-ray diffraction
RP NPs: Red phosphorus nanoparticles
SEM: Scanning electron microscopy
TEM: Transmission electron microscopy
TG: Thermo gravimetric
XPS: X-ray photoelectron spectroscopy
