# Supplementary material for: Facile Solution Synthesis of Red Phosphorus Nanoparticles for Lithium Ion Battery Anodes
Source: Nanoscale Res Lett. 2018 Nov 8;13:356. doi: 10.1186/s11671-018-2770-4 (PMC6223392; doi:10.1186/s11671-018-2770-4)
Supplement: Supplementary file 1 — Figure S1. The reaction process of RPNPs via the solution synthesis. Figure S2. Optical images of RPNPs and commercial RP powders. Figure S3. I-V curves of RPNPs. Figure S4. SEM images of commercial RP. (DOCX 631 kb) [file 11671_2018_2770_MOESM1_ESM.docx]

**Supporting Information**

Facile Solution Synthesis of Red Phosphorus Nanoparticles for Lithium Ion Battery Anodes

Fei Wang, Wenwen Zi, BaoXun Zhao, HongBin Du*

State Key Laboratory of Coordination Chemistry, School of Chemistry and Chemical Engineering, Nanjing University, Nanjing, 210023, China.

***Electronic Supplementary Information contains Figures S1-S4.***


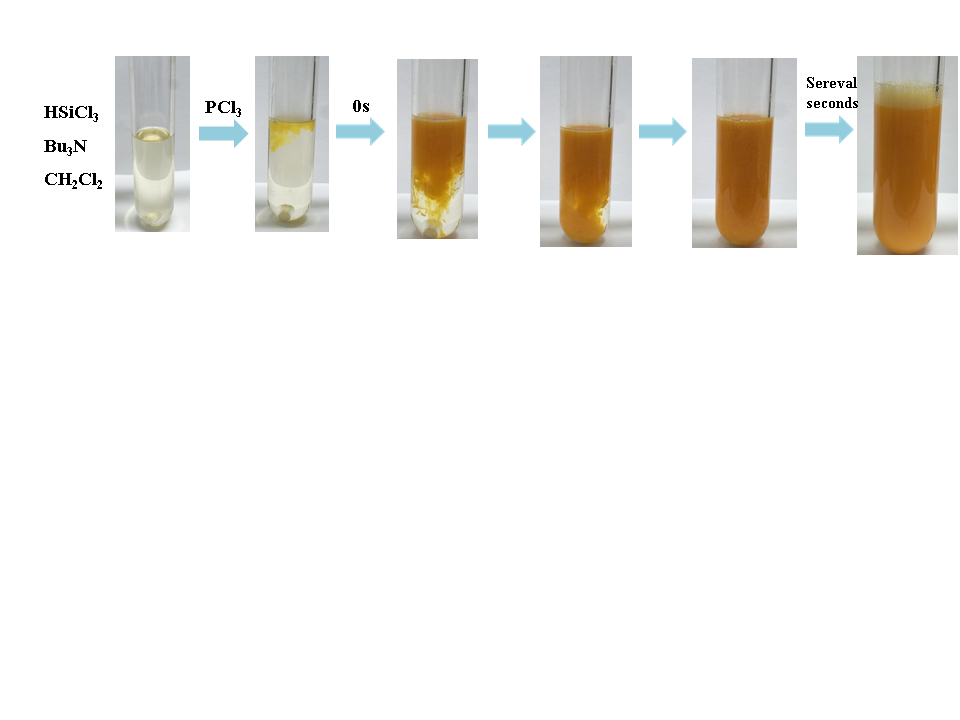


**Figure S1.** The reaction process of RPNPs via the solution synthesis


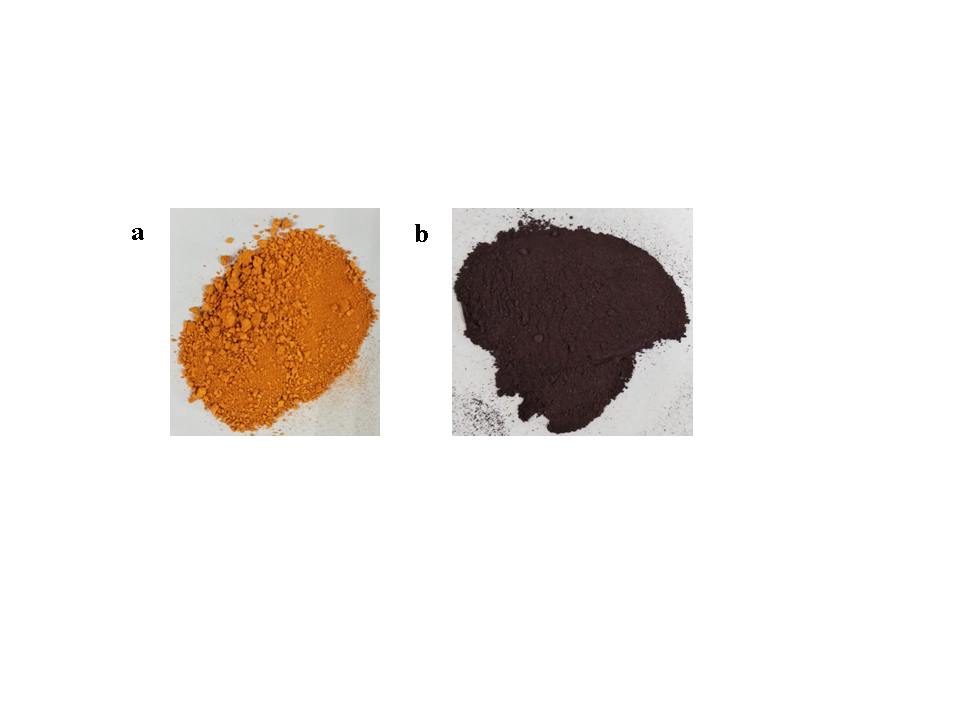


**Figure S2.** Optical images of (a) RPNPs powders and (b) commercial RP powders.





**Figure S3**. I-V curves of RPNPs.


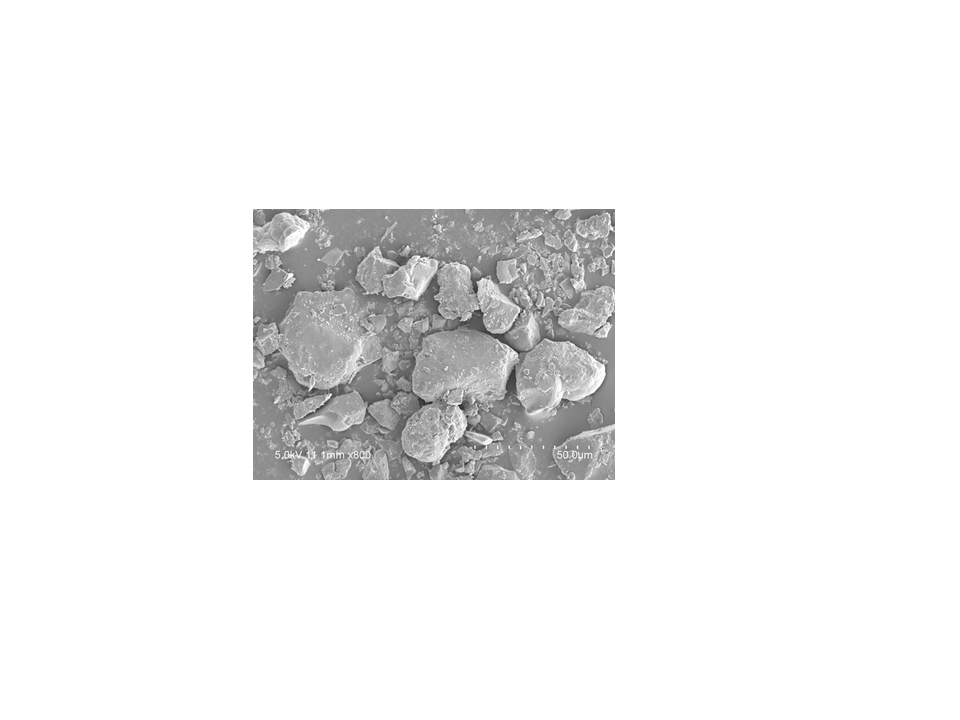


**Figure S4**. SEM image of commercial RP.
